# Microarray Analysis Reveals Higher Gestational Folic Acid Alters Expression of Genes in the Cerebellum of Mice Offspring—A Pilot Study

**DOI:** 10.3390/brainsci5010014

**Published:** 2015-01-26

**Authors:** Subit Barua, Salomon Kuizon, Kathryn K. Chadman, W. Ted Brown, Mohammed A. Junaid

**Affiliations:** 1 Developmental Biochemistry, New York State Institute for Basic Research in Developmental Disabilities, 1050 Forest Hill Road, Staten Island, NY 10314, USA; E-Mails: subitbarua@gmail.com (S.B.); salomon.kuizon@opwdd.ny.gov (S.K.); 2 Developmental Neurobiology, New York State Institute for Basic Research in Developmental Disabilities, 1050 Forest Hill Road, Staten Island, NY 10314, USA; E-Mail: kathryn.chadman@opwdd.ny.gov; 3 Human Genetics, New York State Institute for Basic Research in Developmental Disabilities, 1050 Forest Hill Road, Staten Island, NY 10314, USA; E-Mail: ted.brown@opwdd.ny.gov

**Keywords:** folic acid, DNA methylation, microarray, autism, gene expression, epigenetics, genomic imprinting, prenatal nutrition, brain

## Abstract

Folate is a water-soluble vitamin that is critical for nucleotide synthesis and can modulate methylation of DNA by altering one-carbon metabolism. Previous studies have shown that folate status during pregnancy is associated with various congenital defects including the risk of aberrant neural tube closure. Maternal exposure to a methyl supplemented diet also can alter DNA methylation and gene expression, which may influence the phenotype of offspring. We investigated if higher gestational folic acid (FA) in the diet dysregulates the expression of genes in the cerebellum of offspring in C57BL/6 J mice. One week before gestation and throughout the pregnancy, groups of dams were supplemented with FA either at 2 mg/kg or 20 mg/kg of diet. Microarray analysis was used to investigate the genome wide gene expression profile in the cerebellum from day old pups. Our results revealed that exposure to the higher dose FA diet during gestation dysregulated expression of several genes in the cerebellum of both male and female pups. Several transcription factors, imprinted genes, neuro-developmental genes and genes associated with autism spectrum disorder exhibited altered expression levels. These findings suggest that higher gestational FA potentially dysregulates gene expression in the offspring brain and such changes may adversely alter fetal programming and overall brain development.

## 1. Introduction

The changes in the epigenetic patterning occurring *in utero* may result in alterations in the gene expression and susceptibility to various diseases, which may have a lifelong impact [1,2]. Such changes in DNA methylation and gene expression can be induced by maternal lifestyle and environmental factors including nutrition during the pregnancy. The critical role of folic acid (FA) in the complex of cellular activities was suggested as early as 1944 by the observation that folate deficiency results in an increased incidence of prematurity [3]. Later in 1966 the seminal work by Smithells and Hibbard suggested that folate status during pregnancy is an important contributing factor in the origin of neural tube defects (NTDs) [4,5]. Considering the benefits of FA in reducing the incidence of NTDs, various countries have recommended mandatory fortification of grain and flour since the late 1990’s, and women of childbearing age are recommended to take 400–800 μg/day FA [6,7]. For women with a history of complicated pregnancy, such as a prior NTD-affected baby, the recommended daily FA intake is ten times higher, 4000 μg/day [8]. However, in addition to the recommended FA intake through fortified foods and prescription multivitamin tablets, intake of FA through other sources, such as over the counter vitamins and energy drinks can lead to even higher levels FA exposure. Concern has been raised regarding adverse potential health outcomes of these high levels of FA [5,9]. Recent studies in animal models have shown that exposure to higher FA levels during gestation can induce alterations in methylation and gene expression in the cerebral hemispheres in offspring brain. Some of the genes altered are associated with neural development [10]. Since a dysfunctional folate-methionine pathway has been implicated in many individuals with autism [11], dysregulation in gene expression induced by higher gestational FA may have a profound impact on neural functioning in the offspring.

In addition to cerebral circuits, defects in cerebellar circuits have also been proposed as being involved with autism [12], and such changes in cerebellar circuitry may impair cognition and emotion resulting in autism spectrum disorders (ASDs) [13]. Furthermore, a recent study reported that individuals with autism have reduced number and density of Purkinje cells in the cerebellum compared to controls, suggesting a pattern of developmental alteration in the cerebellum may contribute to the etiology of autism [14]. In order to reveal the clinical significance of the higher FA intake in developing brain, in this pilot study, we conducted a genome wide microarray analysis to evaluate the effect of increased gestational FA on gene expression in the cerebellum of offspring.

## 2. Results

### 2.1. Higher Maternal FA during Gestation Alters Expression of Several Genes in the Cerebellum of Offspring

**Figure 1:**
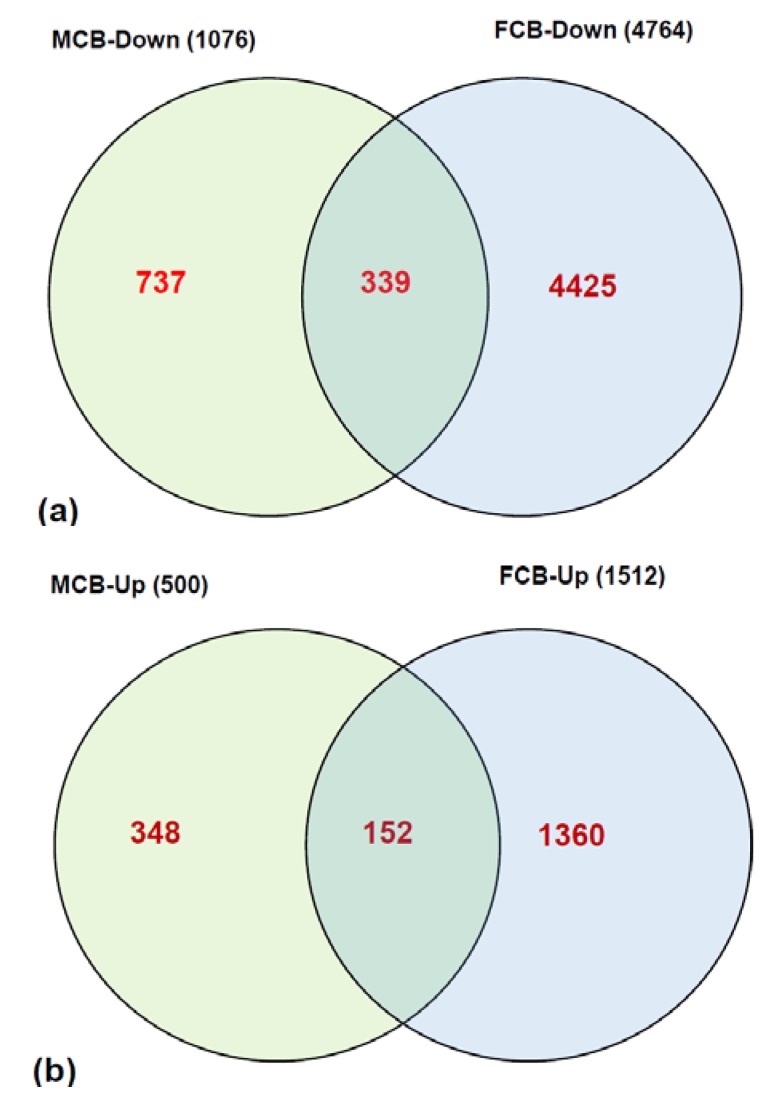
Overview of differential gene expression in the cerebellum of P1 pups. The distribution of (**a**) down-regulated genes, as well as (**b**) up-regulated genes, between male (MCB) and female (FCB) pups from higher gestational FA.

In the microarray analysis of transcripts from 2 mg FA group *vs.* 20 mg FA group of postnatal day one (P1) pups, the expression pattern of a significant number of genes were found to be altered by ≥2.5 fold at a significance of *p* > 0.05. Overall, in male pups the expression patterns of 1076 transcripts were down-regulated (Supplementary Table S1) and 499 transcripts were up-regulated (Supplementary Table S2) in the cerebellum from the 20 mg maternal FA group. In female pups, the expressions of 4764 transcripts (Supplementary Table S3) were down-regulated and 1511 transcripts (Supplementary Table S4) were up-regulated. The expression of 339 transcripts that were down-regulated and 152 transcripts that were up-regulated by ≥2.5 fold in pups’ cerebellum were common for both the genders in the 20 mg maternal FA group (Figure 1). These results of microarray data illustrate that higher maternal FA during gestation can induce significant alterations in the expression of transcripts in offspring brain cerebellum. The data further suggests that in addition to the FA dose, there is an interaction between maternal FA dose and its role in modulating the gene expression levels depending on the sex of the offspring.

### 2.2. Higher Maternal FA Alters Expression of Several Transcription Factors and Imprinted Genes in the Cerebellum of Offspring

Analyses of our data have revealed that a higher intake of maternal FA altered the expression levels of several transcription factors and imprinted genes in the offspring cerebellum (Supplementary Tables S1–S4). Transcriptional regulators such as forkhead box or homeobox genes *Foxa1*, *Foxa2*, *Hoxc11*, *Lhx6* in male pups and *Foxc2*, *Foxf1a*, *Hoxa3*, *Hoxa4*, *Hoxa5* in female pups were altered by ≥2.5 fold. Though the direction of expression changes varied between male and female pups, some transcription factors (*Esr1*, *Sp3*, *Sqstm1* and *Zfp93*) altered were common in both the genders from the higher maternal FA group.

Further analysis of our data revealed dysregulation in the expression of several imprinted genes in both male and female pups from the higher maternal FA group. In male pups, the expression of imprinted genes *Kcnq1*, *Tsix* were down-regulated, while the expression of *Snrpn* was up-regulated. In contrast, in female pups while the expression of imprinted genes *Mirg* and *Rhox5* were up-regulated, several genes including *H19*, *H12*, *Peg12*, *Peg3* and *Xist* were down regulated by ≥2.5 fold. Since transcription factors and imprinted genes play a key role in developmental outcomes, such dysregulation in gene expression as a result of higher gestational FA may have adverse outcomes in overall development of the offspring cerebellum.

### 2.3. Higher Maternal FA Alters the Expression Profile of Several Neural Genes in the Cerebellum of Offspring

In order to study the effect of higher gestational FA on gene expression at a functional level, we analyzed the differential expression of neural genes and grouped them based on their functional role in specific neural pathways (Table 1 and Table 2). We found higher amounts of gestational FA altered expression of several neural genes in both male and female pups. The extent of variation was between 6-fold down-regulation in males and 13-fold down-regulation in females. In male pups from the higher maternal FA group, the expression of genes relating to circadian rhythm (*Cry1*), dopaminergic and serotonergic pathways (*Slc18a2*, *Pde4c*, *Slc6a4*, *Pde4b*), gap junctions (*Hras1*, *Tubb3*, *Tubb2b*), neurogenesis (*Hes1*, *Rac1*), including genes related to neuronal-ion channel, neurotrophin-receptor and synaptic plasticity exhibited dysregulation in expression. In female pups from the higher gestational FA group, the expression of several genes relating to circadian rhythm (*Prkar2b*, *Smad4*, *Bhlhe41*, *Mtnr1b*, *Ncoa3*), dopaminergic and serotonergic pathways (*Dusp1*, *Pde10a*, *Fos*), GABA-glutamate pathway (*Avp*, *Cacna1a*, *Gabrb1*, *Grin1*, *Slc1a6*), gap junctions (*Kras*, *Map2k1*, *Gja5*, *Cav1*, *Gja3*), neurogenesis (*Vegfa*, *Ntn1*, *Dvl3*, *Robo1*, *Pax5*, *Stat3*), including neurotransmitter, neuronal-ion channel, neurotrophin-receptor and synaptic plasticity were dysregulated significantly. Further analysis of the data revealed several neural genes (*Cdk1*, *Csnk1a1*, *Frs3*, *Nsf* and *Tuba3a*) were down-regulated, and genes related to the dopaminergic and serotonergic pathway (*Adcy3*) were up-regulated in both male and female pups from the higher maternal FA group. Thus our data suggests that higher maternal FA significantly alters expression of several neural genes in the cerebellum of offspring, and some of these changes varied in a gender specific manner.

**Table 1:** Neural genes altered ≥2.5 fold in the cerebellum of male pups from mothers having FA supplementation during gestation at 20 mg/kg in comparison to mothers at 2 mg/kg diet.

Accession	Symbol	Fold Change	Direction of Change	Pathway
ref|NM_146087	Csnk1a1	−3.90	↓	Circadian
ref|NM_007771	Cry1	−2.70	↓	Circadian
ref|NM_172523	Slc18a2	−3.81	↓	Dopa-Serotonin
ref|NM_201607	Pde4c	−3.11	↓	Dopa-Serotonin
ref|NM_010484	Slc6a4	−3.06	↓	Dopa-Serotonin
ref|NM_008740	Nsf	−3.40	↓	GABA-glutamate
ref|NM_001130444	Hras1	−4.63	↓	Gap-Junctions
ref|NM_009446	Tuba3a	−3.17	↓	Gap-Junctions
ref|NM_023279	Tubb3	−2.76	↓	Gap-Junctions
ref|NM_023716	Tubb2b	−2.71	↓	Gap-Junctions
ref|NM_007659	Cdk1	−2.53	↓	Gap-Junctions
ref|NM_008235	Hes1	−3.17	↓	Neurogenesis
ref|NM_001039104	Trpm1	−3.36	↓	Neuronal-Ion channel
ens|ENSMUST00000105918	Kcnq1	−3.29	↓	Neuronal-Ion channel
ref|NM_144939	Frs3	−6.24	↓	Neurotrophin-receptor
ref|NM_053075	Rheb	−3.13	↓	Synaptic Plasticity
ref|NM_138305	Adcy3	2.71	↑	Dopa-Serotonin
ref|NM_019840	Pde4b	2.57	↑	Dopa-Serotonin
ref|NM_009007	Rac1	2.73	↑	Neurogenesis
ref|NM_011913	Best1	2.62	↑	Neuronal-Ion channel
ref|NM_009314	Tacr2	2.73	↑	Neurotransmitter
ref|NM_009750	Ngfrap1	2.92	↑	Neurotrophin-receptor

↓ = Down regulated; ↑ = Up regulated.

**Table 2:** Neural genes altered ≥2.5 fold in the cerebellum of female pups from mothers having FA supplementation during gestation at 20 mg/kg in comparison to mothers at 2 mg/kg diet.

Accession	Symbol	Fold Change	Direction of Change	Pathway
ref|NM_146087	Csnk1a1	−7.80	↓	Circadian
ref|NM_011158	Prkar2b	−6.84	↓	Circadian
ref|NM_013672	Sp1	−5.80	↓	Circadian
ref|NM_008540	Smad4	−5.02	↓	Circadian
ref|NM_172563	Hlf	−4.27	↓	Circadian
ref|NM_009602	Chrnb2	−4.10	↓	Circadian
ref|NM_001174053	Camk2b	−3.37	↓	Circadian
ref|NM_015822	Fbxl3	−3.27	↓	Circadian
ref|NM_009516	Wee1	−3.05	↓	Circadian
ref|NM_010098	Opn3	−3.04	↓	Circadian
ref|NM_008904	Ppargc1a	−2.90	↓	Circadian
ref|NM_011144	Ppara	−2.56	↓	Circadian
ref|NM_013642	Dusp1	−4.63	↓	Dopa-Serotonin
ref|NM_011866	Pde10a	−4.55	↓	Dopa-Serotonin
ref|NM_010234	Fos	−4.47	↓	Dopa-Serotonin
ref|NM_001131020	Gfap	−4.29	↓	Dopa-Serotonin
ref|NM_001012765	Adcy5	−3.83	↓	Dopa-Serotonin
ref|NM_172778	Maob	−3.67	↓	Dopa-Serotonin
ref|NM_001111015	Syn2	−3.11	↓	Dopa-Serotonin
gb|AK032648	Pde4d	−2.63	↓	Dopa-Serotonin
ref|NM_019827	Gsk3b	−2.55	↓	Dopa-Serotonin
ref|NM_009732	Avp	−13.07	↓	GABA-glutamate
ref|NM_007578	Cacna1a	−8.80	↓	GABA-glutamate
ref|NM_008069	Gabrb1	−7.02	↓	GABA-glutamate
ref|NM_146072	Grik1	−5.57	↓	GABA-glutamate
ref|NM_001037724	Adcy7	−5.50	↓	GABA-glutamate
ref|NM_008174	Grm8	−5.34	↓	GABA-glutamate
ref|NM_008171	Grin2b	−5.07	↓	GABA-glutamate
ref|NM_001039195	Gria2	−5.07	↓	GABA-glutamate
ref|NM_001146311	Cln3	−5.01	↓	GABA-glutamate
ref|NM_001013385	Grm4	−4.57	↓	GABA-glutamate
ref|NM_176942	Gabra5	−4.20	↓	GABA-glutamate
ref|NM_010251	Gabra4	−4.12	↓	GABA-glutamate
ref|NM_011393	Slc1a2	−3.99	↓	GABA-glutamate
ref|NM_001113383	Gls	−3.95	↓	GABA-glutamate
ref|NM_182959	Slc17a8	−3.91	↓	GABA-glutamate
ref|NM_016886	Gria3	−3.89	↓	GABA-glutamate
ref|NM_008075	Gabrr1	−3.83	↓	GABA-glutamate
ref|NM_080853	Slc17a6	−3.49	↓	GABA-glutamate
ref|NM_175328	Slc6a15	−3.15	↓	GABA-glutamate
ref|NM_001143834	Grm5	−3.10	↓	GABA-glutamate
ref|NM_008740	Nsf	−3.09	↓	GABA-glutamate
ref|NM_019691	Gria4	−3.03	↓	GABA-glutamate
ref|NM_001042451	Snca	−2.80	↓	GABA-glutamate
ref|NM_147176	Homer1	−2.69	↓	GABA-glutamate
ref|NM_021284	Kras	−6.85	↓	Gap-Junctions
ref|NM_009446	Tuba3a	−6.70	↓	Gap-Junctions
ref|NM_008927	Map2k1	−4.92	↓	Gap-Junctions
ref|NM_010937	Nras	−4.37	↓	Gap-Junctions
ref|NM_011101	Prkca	−3.98	↓	Gap-Junctions
ref|NM_007659	Cdk1	−3.84	↓	Gap-Junctions
ref|NM_001080971	Tubb1	−3.67	↓	Gap-Junctions
ref|NM_011100	Prkacb	−3.64	↓	Gap-Junctions
ref|NM_009447	Tuba4a	−3.62	↓	Gap-Junctions
ref|NM_010288	Gja1	−3.52	↓	Gap-Junctions
ref|NM_175452	Gjc2	−2.91	↓	Gap-Junctions
ref|NM_010930	Nov	−2.91	↓	Gap-Junctions
ref|NM_007912	Egfr	−2.71	↓	Gap-Junctions
ref|NM_009231	Sos1	−2.70	↓	Gap-Junctions
ref|NM_028751	Tjap1	−2.53	↓	Gap-Junctions
ref|NM_001025250	Vegfa	−6.08	↓	Neurogenesis
ref|NM_008744	Ntn1	−5.16	↓	Neurogenesis
ref|NM_007889	Dvl3	−4.83	↓	Neurogenesis
ref|NM_010894	Neurod1	−4.13	↓	Neurogenesis
ref|NM_008781	Pax3	−4.07	↓	Neurogenesis
ref|NM_177821	Ep300	−3.87	↓	Neurogenesis
ref|NM_011443	Sox2	−3.54	↓	Neurogenesis
ref|NM_009599	Ache	−3.50	↓	Neurogenesis
ref|NM_033620	Pard3	−3.34	↓	Neurogenesis
ref|NM_010928	Notch2	−3.25	↓	Neurogenesis
ref|NM_008737	Nrp1	−3.07	↓	Neurogenesis
ref|NM_007553	Bmp2	−2.86	↓	Neurogenesis
ref|NM_022312	Tnr	−2.85	↓	Neurogenesis
ref|NM_010883	Ndp	−2.62	↓	Neurogenesis
ref|NM_001039934	Mtap2	−2.60	↓	Neurogenesis
ref|NM_007865	Dll1	−2.59	↓	Neurogenesis
ref|NM_008973	Ptn	−2.54	↓	Neurogenesis
ref|NM_008421	Kcnc1	−10.41	↓	Neuronal-Ion channel
ref|NM_010597	Kcnab1	−5.07	↓	Neuronal-Ion channel
ref|NM_009900	Clcn2	−3.81	↓	Neuronal-Ion channel
ref|NM_001025581	Kcnc2	−3.45	↓	Neuronal-Ion channel
ref|NM_001083616	Cacna1d	−3.26	↓	Neuronal-Ion channel
ref|NM_008226	Hcn2	−3.23	↓	Neuronal-Ion channel
ref|NM_011930	Clcn7	−3.04	↓	Neuronal-Ion channel
ref|NM_010408	Hcn1	−2.85	↓	Neuronal-Ion channel
ref|NM_145983	Kcna5	−2.67	↓	Neuronal-Ion channel
ref|NM_001099298	Scn2a1	−2.60	↓	Neuronal-Ion channel
ref|NM_001044308	Cacna1i	−2.59	↓	Neuronal-Ion channel
ref|NM_018732	Scn3a	−2.51	↓	Neuronal-Ion channel
ref|NM_010610	Kcnma1	−2.50	↓	Neuronal-Ion channel
ref|NM_153087	Hrh4	−4.01	↓	Neurotransmitter
ref|NM_001081147	Oxtr	−3.68	↓	Neurotransmitter
ref|NM_008285	Hrh1	−2.90	↓	Neurotransmitter
ref|NM_021382	Tacr3	−2.71	↓	Neurotransmitter
ref|NM_009313	Tacr1	−2.70	↓	Neurotransmitter
ref|NM_008747	Ntsr2	−2.61	↓	Neurotransmitter
ref|NM_009219	Sstr4	−2.61	↓	Neurotransmitter
ref|NM_008177	Grpr	−2.53	↓	Neurotransmitter
ref|NM_001025074	Ntrk2	−7.28	↓	Neurotrophin-receptor
ref|NM_009365	Tgfb1i1	−6.21	↓	Neurotrophin-receptor
ref|NM_144939	Frs3	−5.29	↓	Neurotrophin-receptor
ref|NM_011640	Trp53	−4.40	↓	Neurotrophin-receptor
ref|NM_010560	Il6st	−4.00	↓	Neurotrophin-receptor
ref|NM_019791	Maged1	−3.69	↓	Neurotrophin-receptor
ens|ENSMUST00000052164	Ppyr1	−3.47	↓	Neurotrophin-receptor
ref|NM_009911	Cxcr4	−3.39	↓	Neurotrophin-receptor
ref|NM_022024	Gmfg	−3.10	↓	Neurotrophin-receptor
ref|NM_010849	Myc	−3.06	↓	Neurotrophin-receptor
ref|NM_139149	Fus	−2.84	↓	Neurotrophin-receptor
ref|NM_177410	Bcl2	−2.64	↓	Neurotrophin-receptor
ref|NM_001025074	Ntrk2	−7.28	↓	Synaptic Plasticity
ref|NM_028736	Grip1	−6.77	↓	Synaptic Plasticity
ref|NM_020493	Srf	−3.25	↓	Synaptic Plasticity
ens|ENSMUST00000111939	Nos1	−2.97	↓	Synaptic Plasticity
ref|NM_013498	Crem	−2.88	↓	Synaptic Plasticity
ref|NM_009952	Creb1	−2.83	↓	Synaptic Plasticity
ref|NM_024469	Bhlhe41	5.46	↑	Circadian
ref|NM_145712	Mtnr1b	4.18	↑	Circadian
ref|NM_008679	Ncoa3	3.70	↑	Circadian
ref|NM_017376	Tef	3.48	↑	Circadian
ref|NM_011281	Rorc	3.19	↑	Circadian
ref|NM_138305	Adcy3	4.41	↑	Dopa-Serotonin
ref|NM_008169	Grin1	5.56	↑	GABA-glutamate
ref|NM_009200	Slc1a6	2.80	↑	GABA-glutamate
ref|NM_008121	Gja5	4.27	↑	Gap-Junctions
ref|NM_007616	Cav1	3.57	↑	Gap-Junctions
ref|NM_016975	Gja3	2.98	↑	Gap-Junctions
ref|NM_011840	Map2k5	2.72	↑	Gap-Junctions
ref|NM_008361	Il1b	2.67	↑	GABA-glutamate
ens|ENSMUST00000114274	Robo1	4.31	↑	Neurogenesis
ref|NM_008782	Pax5	3.39	↑	Neurogenesis
ref|NM_011486	Stat3	2.92	↑	Neurogenesis
ref|NM_001077403	Nrp2	2.52	↑	Neurogenesis
ref|NM_183000	Accn3	4.44	↑	Neuronal-Ion channel
ref|NM_007699	Chrm4	3.82	↑	Neurotransmitter
ref|NM_013462	Adrb3	2.61	↑	Neurotransmitter
ref|NM_008006	Fgf2	2.65	↑	Neurotrophin-receptor
ref|NM_008416	Junb	2.86	↑	Synaptic Plasticity
ref|NM_007664	Cdh2	2.82	↑	Synaptic Plasticity

↓ = Down regulated; ↑ = Up regulated.

## 3. Discussion

In this study, microarray analysis of gene expression was utilized as a tool to investigate the effect of gestational FA on the transcriptome of cerebellum from P1 pups. Our data has shown that higher maternal FA during gestation dysregulated the expression of several genes that may play a causal role in developmental anomalies. In particular, the expression of several genes that code for genomic imprinting, transcriptional regulation and neural plasticity were significantly altered as a result of higher amounts of maternal FA. Genomic imprinting is a specialized epigenetic phenomenon resulting in monoallelic parent-specific expression of genes due to differential DNA methylation and histone modifications of specific gene regions [15]. Perturbations in dosage compensation can cause developmental issues including changes in growth and behavior [15]. Since the dynamics of genomic imprinting take place during gametogenesis and early embryogenesis; FA, by virtue of being a methyl group modulator during gestation, may play a critical role in affecting DNA methylation in the offspring epigenome. Our results have shown that higher gestational FA dysregulated expression of several imprinted genes in the cerebellum of P1 offspring. For example, in male pups the expression of the gene *Kcnq1*, a voltage-gated potassium channel, which has been associated with Jervell and Lange-Nielsen syndrome and hereditary long QT syndrome 1 [16,17], and the gene *Tsix* that expresses the non-coding antisense transcript required for imprinted X inactivation [18], were down-regulated. In contrast, the expressions of the gene *Snrpn* that plays a role in Angelman syndrome or Prader-Willi syndrome [19] was up-regulated. Similarly, in female pups, the miRNA containing gene (*Mirg*), and reproductive homeobox-5 gene (*Rhox5*), were up-regulated, while the expression of several imprinted genes, including *H13*, *H19*, *Igf2* and *Xist* were down-regulated. Since, a methylation defect at the *Igf2*/*H19* locus can result in imprinting disorders, and has been associated with Beckwith-Wiedemann syndrome [20,21], such gene dysregulation as a result of higher maternal FA may modulate epigenetic abnormalities in the pathogenesis of growth and development.

Next, our analysis further revealed dysregulation in the expression of several transcription factors in the cerebellum of P1 pups from higher maternal FA compared to pups from dams having FA at 2 mg/kg diet. The dysregulation was observed in many genes including the evolutionarily conserved class of homeobox genes in both male and female pups. The expression of several transcription factors such as *Esr1*, *Sp3*, *Zfp93*, *Sqstm1* were dysregulated in pups of both genders from dams having higher gestational FA, though the direction of changes were not always same. Since transcription factors orchestrate gene expression of complex cellular machineries, such gene dysregulation may modulate the development of many organ systems, including the brain.

Higher maternal FA altered expression of several genes involved in dopamine-serotonin, GABA-glutamate, gap-junctions, neuronal-ion channel, neurogenesis, synaptic plasticity and the circadian pathway (Table 1 and Table 2). Higher gestational FA also altered expression of several genes that were earlier shown to be associated with ASDs as well as cognitive development. For example, in male pups from higher gestational FA, the serotonin transporter gene *Slc6a4* that encodes an integral membrane protein, which plays a crucial role in serotonergic neurotransmission [22], was down-regulated by three fold; *Fezf2*, a gene belonging to zinc finger family [23], and *Mbd3* a gene belonging to a family of nuclear proteins having methyl-CpG binding domain [24], were significantly up-regulated more than three fold. Of note, it has been shown that the alterations in the expression of *Slc6a4* can be mediated due to epigenetic modulation in the promoter associated CpG island by environmental factors [25]. Such changes in *Slc6a4* expression have been associated with alterations in brain structure and various neuropsychiatric illnesses including depression [26,27,28]. In female pups, the expression of several genes associated with ASDs (Table 1 and Table 2, Supplementary Tables S3 and S4) e.g., *Fmr1*, *Grid1*, *Nrxn1*, *Grip1* were down-regulated, and *Nrp2*, a gene belonging to the neuropilin family of receptor proteins was up-regulated from dams having higher gestational FA. *Fmr1* has long been associated with developmental cognitive impairment such as fragile X syndrome (FXS) and, found to be accompanied by epigenetic modification along with genetic anomalies [29,30,31]. Earlier, by using lymphoblastoid cells, we have shown that FA supplementation down-regulates the expression of the *FMR1* gene, both at mRNA and protein levels [32]. Moreover, a recent study has suggested strong evidence for multiple medical, cognitive and psychiatric difficulties among women with the *FMR1* premutation [33]. Similarly, when the ionotropic glutamate receptor *Grid1* is deleted, it results in aberrant emotional and social behavior in mice [34]. Here *Grid1* was found to be down-regulated in female pups from dams with higher FA. In contrast, *Nrp2*, a neuropilin family gene that controls neuronal migration and axon guidance, and has been associated with autism, wasup-regulated in female pups from dams having higher gestational FA [35]. Previous studies have shown that pups exposed to higher gestational and post-weaning FA exhibited increased ultrasonic vocalizations, greater anxiety-like behavior and hyperactivity [36]. Indeed, higher gestational FA during gestation altered methylation in the cerebral hemispheres of the offspring in both intergenic and intragenic regions, and thus modulated the expression of several genes [10]. In the current study, we found higher FA dysregulates the expression of several genes including those associated with neural development in the cerebellum of the offspring. Mechanistically, such dysregulations may have been induced by the precise changes in the DNA methylation *in utero*, modulated by higher gestational FA. The dysregulation of gene expression may be due to the direct impact of site or gene specific changes in the promoter or gene body region of specific genes or it may result from distant effects from changes in signaling pathways.

Metabolically, FA (pteroylmonoglutamate) consumed through supplementation or fortified foods is reduced by the enzyme dihydrofolate reductase primarily to dihydrofolate, and chemically reduced to tetrahydrofolate in the liver, where it collects a formyl group which is reduced to methyl to form 5-methyl-tetrahydrofolate [5]. However with the supplementation of high doses of FA this process is swamped by saturation of dihydrofolate reductase activity, and these may result in a considerable increase of unmetabolized FA in plasma [37]. Such evidence was further confirmed by the presence of unmetabolized FA in cord blood [38]. Though the exact mechanism by which this excess maternal FA is modulating the fetal brain development is not known, we speculate it may be a combined effect of pteroylmonoglutamate and methyl-tetrahydrofolate. One possibility is, with higher supplementation of maternal FA, a proportion of high dose FA that enters tissue becomes a substrate for polyglutamate synthase but is inert and thus excess FA in blood may compete with methyl-tetrahydrofolate to bind with the folate receptor [39] in the choroid plexus and inhibit methyl-tetrahydrofolate transport in the developing brain and thus interfere with regulatory functions. Alternatively an increased methyl-folate via maternal metabolism may modulate the methylation profile of imprinted genes, which have key roles in maintaining brain homeostasis and development.

Since, early life experience may alter the brain structure and function including behavioral and neural development, higher gestational FA may have untoward side effects in overall development of the offspring resulting in lifelong effects. The causal link between higher FA supplementation and ASD incidence rates was hypothesized by previous studies [40,41], and a recent study from the Rochester Epidemiological Project in Rochester, MN (1976–1997), found a strong correlation (0.87) of ASD incidence in offspring for maternal consumption of prenatal vitamins containing >1 mg of folic acid [42,43]. Such evidence suggests that while too little FA results in damage to nervous tissue as evident from several studies, too much FA may disrupt normal function of nervous tissue associated with autism. Indeed concern has been raised with the role of excess maternal FA with several developmental outcomes including congenital heart defects, oral clefts, asthma, cancer and ASD [5,44].

**Figure 2:**
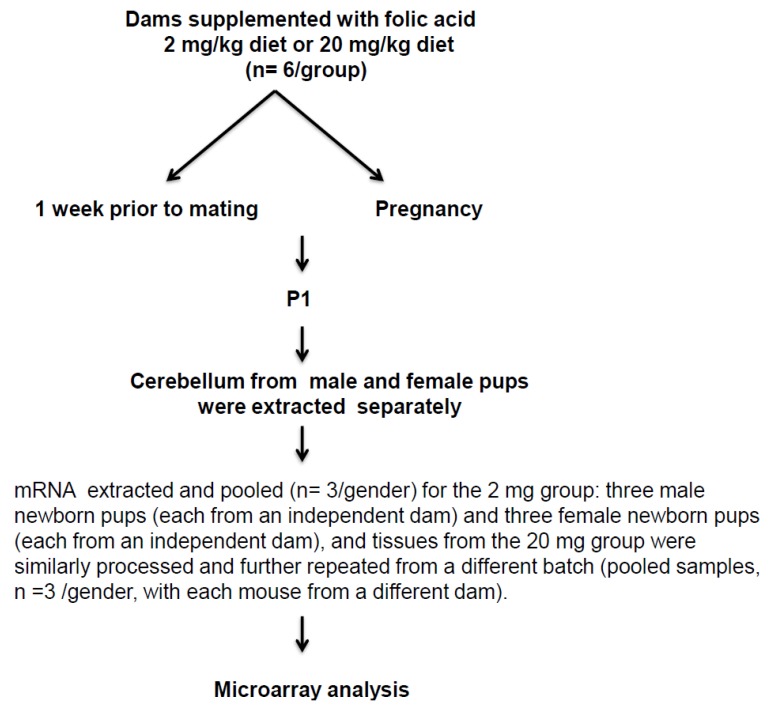
Schematic diagram of the study design illustrating main experimental approach.

While analysis of the microarray data revealed that the expression of a number of genes exhibited the same direction of change in both male and female pups from the higher gestational FA group (Figure 2), the expressions of several other genes were sex biased. Indeed, we found that the expression of genes in the cerebellum of female pups were more vulnerable to the changes induced by higher gestational FA. It shows that the *in utero* exposure of gestational FA may have a different impact depending upon the gender of the progeny, and it may have distinct mechanisms of alterations and consequences. Though the precise mechanism of such changes cannot be established from the present study, it confirms our previous data showing that the gestational FA induces differential alterations in methylation and gene expression in the brain of offspring depending upon gender of the progeny [10]. Of note, several previous studies have shown strong evidence that the susceptibility to neuropsychiatric and neurodegenerative diseases varies between men and women [45,46,47]; and maternal diet can significantly influence fetal outcomes including biasing offspring sex ratio [48]. Moreover, a recent study has shown widespread differences in the splicing and expression of genes in human brain between male and female [49]. Another study has shown that the maturation of *GABAA* signaling follows sex-specific patterns, which correlate with brain developmental expression profiles [50]. The switch from GABA being excitatory to inhibitory occurs earlier in females than in males.

## 4. Material and Methods

### 4.1. Mice Strain and Feeding

Adult, 8- to 10-week-old C57BL/6J mice were used in all the experiments, and handled according to the protocol reviewed and approved by the Institute for Basic Research Institutional Animal Care and Use Committee. One week prior to mating and throughout the pregnancy, female mice were fed with a custom AIN-93G amino acid-based diet (Research Diet, Inc. New-Brunswick, NJ, USA), having FA at 2 mg/kg (*n* = 6) and 20 mg/kg (*n* = 6) diet. While the diet containing 2 mg of FA/kg diet is the general recommended basal dietary requirements for rodents [51]; the level of 20 mg FA/kg diet is tenfold higher than the basal dietary requirements. These amounts were chosen in this study, considering women with previous history of NTD affected pregnancy are recommended ten-fold higher FA (4 mg FA/day) in comparison to normal pregnant women (400–800 μg/day). Studies have shown in human Tolerable Upper Intake Level (1 mg/day adults) becomes limiting and intake of higher than 1mg/day escalates circulating unmetabolized FA [37]. The supplemented dietary FA level used in this study may not accurately reflect the precise level of FA in human, considering the inherent physiological differences in folate metabolism. Indeed, dihydrofolate reductase activity of rodent liver is perhaps higher than in human liver and to determine the impact of systemic exposure to FA and to elicit the same circulating plasma concentration of FA as human, perhaps rodents have to ingest much greater than pro-rata amounts of FA as human [37,52].

At postnatal day one (P1), for 2 mg group: male pups *n* = 3 and female pups *n* = 3 were sacrificed, and the cerebellum tissues were collected. For 20 mg group: male pups *n* = 6 and female pups *n* = 6 were similarly processed. All tissues were rapidly frozen after dissection and stored at −80 °C until downstream analysis was performed. The schematic diagram of the experimental design for this study is shown in Figure 2.

### 4.2. RNA Preparation

Cerebellum tissues of P1 pups were pooled (*n* = 3/gender) for the 2 mg group with three male pups (each from an independent dam) and three female pups (each from an independent dam), for a total of six pups (*n* = 6). Tissues from 20 mg group were similarly processed. RNA extractions for 20 mg group were further repeated from a different batch (pooled samples, *n* = 3/gender, with each mouse from a different dam), for a total of twelve pups (*n* = 12). Total RNA was extracted with Trizol reagent (Life Technologies, Carlsbad, CA, USA) and further purified by Qiagen RNeasy kit (Qiagen, Valencia, CA, USA), according to the manufacturer’s instructions and processed as described earlier [10,36]. All RNA samples were stored at −80 °C until downstream analysis was performed.

### 4.3. Microarrays

To minimize the differences of individual variability and increase the statistical power, microarray analysis was performed using purified RNA from pooled samples segregated by gender for each group as previously described [36]. Total RNA was reverse-transcribed, and cyanine-3-labelled cRNA was generated by following the manufacturer’s instructions using the one-color, low-input QuickAmp labeling kit (Agilent Technologies, Santa Clara, CA, USA). Following purification of cRNA with RNeasy kit (Qiagen, Valencia, CA, USA), the incorporation of cyanine-3 was assessed by a Nano Drop spectrophotometer. For each sample, a total of 600 ng of cRNA was fragmented and hybridized to the SurePrint G3 mouse gene expression 8 × 60 k arrays (G4852A, Agilent Technologies, Santa Clara, CA, USA). Using Feature Extraction software v10.7.3.1, the data were processed for image analysis and initial quality control. Our Minimum Information about a Microarray Experiment (MIAME)—compliant data has been deposited in the Gene Expression Omnibus database of the National Center for Bioinformatics, with the accession number GSE60531.

### 4.4. Data Analysis

Further analyses of microarray data were conducted with the publicly available BRB ArrayTools 4.3.1-stable release as previously described [36,53]. Quantile-Normalization was used to normalize the expression data and log-transformed. The differential expression of transcripts were assessed by following a series of filtering steps based on intensity filter, *p* value of log intensity variation and fold changes. For each spot if the intensity was below the minimum, a threshold to minimum 10 was used. To further filter the data, a cut-off set at *p* > 0.05 and percent missing exceeds 50% was used to exclude the corresponding spots for an entire gene from all arrays having variation in log intensity. Following filtering, a transcript was considered as differentially expressed if the difference in the mean expression in between two groups was ≥2.5 fold.

## 5. Conclusions

The role of FA in the prevention of NTDs has been well established for several years. However, considering the key role of FA in one-carbon metabolism and modulating the methylation profile, excess FA during gestation may alter the DNA methylation and gene expression of offspring. In this pilot study, we employed genome-wide profiling and found that higher gestational FA altered gene expression of several imprinted genes, transcription factors and genes associated with various neural pathways, including those reported to impact autism etiology, in the cerebellum of the offspring. In addition, several genes were dysregulated differentially in a gender-biased manner in pups from higher gestational FA. Since the cerebellum plays an important role to maintain abundant connections with non-motor brain regions [12], such dysregulations in the expression of genes associated with autism and neurodevelopment may influence overall cognitive development. In light of a recent report that higher FA intake can moderately modify the behavior of offspring [36,54], we suggest moderation of FA supplementation be exercised during gestation. Indeed, considering the shared metabolism between vitamin B12 and folate, high FA supplementation may mask vitamin B12 deficiency in pregnant woman and thus can have adverse consequences in the health of their children [55,56]. Since, vitamin B12 plays an important role in myelination and brain myelination is concentrated from mid-gestation, such imbalance in metabolism may result in impairment in early brain development [57].

This study was conducted as part of a pilot study to elucidate the overall difference in the transcriptional profile as a result of higher gestational FA in the cerebellum of offspring. One of the limitations of our current study is the limited sample size. Moreover as autism is a wider spectrum of disorders not a single entity and now diagnosis is more refined, caution has to be taken in relating findings in other species to humans as biochemical pathways may concur but have different rate limiting determinants. In the future, additional biochemical studies including behavioral analysis with larger numbers of samples will help to more precisely determine the influence of gestational FA in neural disease and would make it a fertile ground for exploration of ASDs.

## Supplementary Information

**Table S1.** Genes down-regulated ≥2.5 fold in the cerebellum of male pups from mothers having FA supplementation during gestation at 20 mg/kg in comparison to mothers at 2 mg/kg diet.

**Table S2.** Genes up-regulated ≥2.5 fold in the cerebellum of male pups from mothers having FA supplementation during gestation at 20 mg/kg in comparison to mothers at 2 mg/kg diet.

**Table S3.** Genes down-regulated ≥2.5 fold in the cerebellum of female pups from mothers having FA supplementation during gestation at 20 mg/kg in comparison to mothers at 2 mg/kg diet.

**Table S4.** Genes up-regulated ≥2.5 fold in the cerebellum of female pups from mothers having FA supplementation during gestation at 20 mg/kg in comparison to mothers at 2 mg/kg diet.

## References

[1] Heijmans B.T., Tobi E.W., Stein A.D., Putter H., Blauw G.J., Susser E.S., Slagboom P.E., Lumey L.H. (2008). Persistent epigenetic differences associated with prenatal exposure to famine in humans. Proc. Natl. Acad. Sci. USA.

[2] Vanhees K., Vonhogen I.G., van Schooten F.J., Godschalk R.W. (2014). You are what you eat, and so are your children: The impact of micronutrients on the epigenetic programming of offspring. Cell Mol. Life Sci..

[3] Callender S. (1944). A critical review of pernicious anaemia of pregnancy. Q. J. Med..

[4] Hibbard E.D., Smithells R.W. (1965). Folic acid metabolism and human embryopathy. Lancet.

[5] Barua S., Kuizon S., Junaid M.A. (2014). Folic acid supplementation in pregnancy and implications in health and disease. J. Biomed. Sci..

[6] Honein M.A., Paulozzi L.J., Mathews T.J., Erickson J.D., Wong L.Y. (2001). Impact of folic acid fortification of the US food supply on the occurrence of neural tube defects. JAMA.

[7] De Wals P., Tairou F., van Allen M.I., Uh S.H., Lowry R.B., Sibbald B., Evans J.A., van den Hof M.C., Zimmer P., Crowley M. (2007). Reduction in neural-tube defects after folic acid fortification in Canada. N. Engl. J. Med..

[8] U.S. Preventive Services Task Force (2009). Folic acid for the prevention of neural tube defects: U.S. Preventive Services Task Force recommendation statement. Ann. Intern. Med..

[9] Choumenkovitch S.F., Selhub J., Wilson P.W., Rader J.I., Rosenberg I.H., Jacques P.F. (2002). Folic acid intake from fortification in United States exceeds predictions. J. Nutr..

[10] Barua S., Kuizon S., Chadman K.K., Flory M.J., Brown W.T., Junaid M.A. (2014). Single-base resolution of mouse offspring brain methylome reveals epigenome modifications caused by gestational folic acid. Epigenetics Chromatin.

[11] Main P.A., Angley M.T., Thomas P., O’Doherty C.E., Fenech M. (2010). Folate and methionine metabolism in autism: A systematic review. Am. J. Clin. Nutr..

[12] Wang S.S., Kloth A.D., Badura A. (2014). The Cerebellum, Sensitive Periods, and Autism. Neuron.

[13] Reeber S.L., Otis T.S., Sillitoe R.V. (2013). New roles for the cerebellum in health and disease. Front. Syst. Neurosci..

[14] Wegiel J., Flory M., Kuchna I., Nowicki K., Ma S., Imaki H., Wegiel J., Cohen I.L., London E., Wisniewski T., Brown W. (2014). Stereological study of the neuronal number and volume of 38 brain subdivisions of subjects diagnosed with autism reveals significant alterations restricted to the striatum, amygdala and cerebellum. Acta Neuropathol. Commun..

[15] Horsthemke B. (2014). In brief: Genomic imprinting and imprinting diseases. J. Pathol..

[16] Tranebjaerg L., Bathen J., Tyson J., Bitner-Glindzicz M. (1999). Jervell and Lange-Nielsen syndrome: A Norwegian perspective. Am. J. Med. Genet..

[17] Crotti L., Celano G., Dagradi F., Schwartz P.J. (2008). Congenital long QT syndrome. Orphanet J. Rare Dis..

[18] Senner C.E., Brockdorff N. (2009). Xist gene regulation at the onset of X inactivation. Curr. Opin. Genet. Dev..

[19] Plagge A. (2012). Non-Coding RNAs at the Gnas and Snrpn-Ube3a Imprinted Gene Loci and Their Involvement in Hereditary Disorders. Front. Genet..

[20] Riccio A., Sparago A., Verde G., de Crescenzo A., Citro V., Cubellis M.V., Ferrero G.B., Silengo M.C., Russo S., Larizza L., Cerrato F. (2009). Inherited and Sporadic Epimutations at the IGF2-H19 locus in Beckwith-Wiedemann syndrome and Wilms’ tumor. Endocr. Dev..

[21] Soejima H., Higashimoto K. (2013). Epigenetic and genetic alterations of the imprinting disorder Beckwith-Wiedemann syndrome and related disorders. J. Hum. Genet..

[22] Antypa N., Serretti A., Rujescu D. (2013). Serotonergic genes and suicide: A systematic review. Eur. Neuropsychopharmacol..

[23] Kwan K.Y. (2013). Transcriptional dysregulation of neocortical circuit assembly in ASD. Int. Rev. Neurobiol..

[24] Cukier H.N., Rabionet R., Konidari I., Rayner-Evans M.Y., Baltos M.L., Wright H.H., Abramson R.K., Martin E.R., Cuccaro M.L., Pericak-Vance M.A., Gilbert J.R. (2010). Novel variants identified in methyl-CpG-binding domain genes in autistic individuals. Neurogenetics.

[25] Vijayendran M., Beach S.R., Plume J.M., Brody G.H., Philibert R.A. (2012). Effects of genotype and child abuse on DNA methylation and gene expression at the serotonin transporter. Front. Psychiatry.

[26] Huang C.H., Santangelo S.L. (2008). Autism and serotonin transporter gene polymorphisms: A systematic review and meta-analysis. Am. J. Med. Genet. B Neuropsychiatr. Genet..

[27] Oberlander T.F. (2012). Fetal serotonin signaling: Setting pathways for early childhood development and behavior. J. Adolesc. Health.

[28] Dannlowski U., Kugel H., Redlich R., Halik A., Schneider I., Opel N., Grotegerd D., Schwarte K., Schettler C., Ambree O. (2014). Serotonin transporter gene methylation is associated with hippocampal gray matter volume. Hum. Brain Map..

[29] Tabolacci E., Chiurazzi P. (2013). Epigenetics, fragile X syndrome and transcriptional therapy. Am. J. Med. Genet. A.

[30] Brown W.T. (2012). Clinical aspects of the fragile X syndrome. Results Probl. Cell Differ..

[31] Tsiouris J.A., Brown W.T. (2004). Neuropsychiatric symptoms of fragile X syndrome: Pathophysiology and pharmacotherapy. CNS Drugs.

[32] Junaid M.A., Kuizon S., Cardona J., Azher T., Murakami N., Pullarkat R.K., Brown W.T. (2011). Folic acid supplementation dysregulates gene expression in lymphoblastoid cells—Implications in nutrition. Biochem. Biophys. Res. Commun..

[33] Wheeler A.C., Bailey D.B., Berry-Kravis E., Greenberg J., Losh M., Mailick M., Mila M., Olichney J.M., Rodriguez-Revenga L., Sherman S. (2014). Associated features in females with an FMR1 premutation. J. Neurodev. Disord..

[34] Yadav R., Gupta S.C., Hillman B.G., Bhatt J.M., Stairs D.J., Dravid S.M. (2012). Deletion of glutamate delta-1 receptor in mouse leads to aberrant emotional and social behaviors. PLoS One.

[35] Wu S., Yue W., Jia M., Ruan Y., Lu T., Gong X., Shuang M., Liu J., Yang X., Zhang D. (2007). Association of the neuropilin-2 (NRP2) gene polymorphisms with autism in Chinese Han population. Am. J. Med. Genet. B Neuropsychiatr. Genet..

[36] Barua S., Chadman K.K., Kuizon S., Buenaventura D., Stapley N.W., Ruocco F., Begum U., Guariglia S.R., Brown W.T., Junaid M.A. (2014). Increasing Maternal or Post-Weaning Folic Acid Alters Gene Expression and Moderately Changes Behavior in the Offspring. PLoS One.

[37] Bailey S.W., Ayling J.E. (2009). The extremely slow and variable activity of dihydrofolate reductase in human liver and its implications for high folic acid intake. Proc. Natl. Acad. Sci. USA.

[38] Sweeney M.R., McPartlin J., Weir D.G., Daly S., Pentieva K., Daly L., Scott J.M. (2005). Evidence of unmetabolised folic acid in cord blood of newborn and serum of 4-day-old infants. Br. J. Nutr..

[39] Smith A.D., Kim Y.I., Refsum H. (2008). Is folic acid good for everyone?. Am. J. Clin. Nutr..

[40] Rogers E.J. (2008). Has enhanced folate status during pregnancy altered natural selection and possibly Autism prevalence? A closer look at a possible link. Med. Hypotheses.

[41] Leeming R.J., Lucock M. (2009). Autism: Is there a folate connection?. J. Inherit. Metab. Dis..

[42] Beard C.M., Panser L.A., Katusic S.K. (2011). Is excess folic acid supplementation a risk factor for autism?. Med. Hypotheses.

[43] O’Neill R.J., Vrana P.B., Rosenfeld C.S. (2014). Maternal methyl supplemented diets and effects on offspring health. Front. Genet..

[44] Neggers Y.H. (2014). Increasing prevalence, changes in diagnostic criteria, and nutritional risk factors for autism spectrum disorders. ISRN Nutr..

[45] Cahill L. (2006). Why sex matters for neuroscience. Nat. Rev. Neurosci..

[46] Cosgrove K.P., Mazure C.M., Staley J.K. (2007). Evolving knowledge of sex differences in brain structure, function, and chemistry. Biol. Psychiatry.

[47] Jazin E., Cahill L. (2010). Sex differences in molecular neuroscience: From fruit flies to humans. Nat. Rev. Neurosci..

[48] Rosenfeld C.S. (2011). Periconceptional influences on offspring sex ratio and placental responses. Reprod. Fertil. Dev..

[49] Trabzuni D., Ramasamy A., Imran S., Walker R., Smith C., Weale M.E., Hardy J., Ryten M. (2013). Widespread sex differences in gene expression and splicing in the adult human brain. Nat. Commun..

[50] Galanopoulou A.S. (2008). Sexually dimorphic expression of KCC2 and GABA function. Epilepsy Res..

[51] Reeves P.G., Nielsen F.H., Fahey G.C. (1993). AIN-93 purified diets for laboratory rodents: Final report of the American Institute of Nutrition ad hoc writing committee on the reformulation of the AIN-76A rodent diet. J. Nutr..

[52] Deghan M.S., Ishiguro L., Sohn K.J., Medline A., Renlund R., Croxford R., Kim Y.I. (2014). Folic acid supplementation promotes mammary tumor progression in a rat model. PLoS One.

[53] Simon R., Lam A., Li M.C., Ngan M., Menenzes S., Zhao Y. (2007). Analysis of gene expression data using BRB-Array Tools. Cancer Inform..

[54] Shorter K.R., Anderson V., Cakora P., Owen A., Lo K., Crossland J., South A.C., Felder M.R., Vrana P.B. (2014). Pleiotropic effects of a methyl donor diet in a novel animal model. PLoS One.

[55] Dwarkanath P., Barzilay J.R., Thomas T., Thomas A., Bhat S., Kurpad A.V. (2013). High folate and low vitamin B-12 intakes during pregnancy are associated with small-for-gestational age infants in South Indian women: A prospective observational cohort study. Am. J. Clin. Nutr..

[56] Smith A.D. (2007). Folic acid fortification: The good, the bad, and the puzzle of vitamin B-12. Am. J. Clin. Nutr..

[57] Black M.M. (2008). Effects of vitamin B12 and folate deficiency on brain development in children. Food Nutr. Bull..

